# Central nervous system involvement in Whipple's disease: Report of a rare pathological entity and comparative review of treatment strategies and outcomes

**DOI:** 10.1016/j.radcr.2024.11.045

**Published:** 2024-12-19

**Authors:** Moustafa A. Mansour, Reem W. Malaeb, Islam E. Elnemr, Mohamed Abdel-Fattah El-Salamoni, Hamdi Nabawi Mostafa, Zarina Ahmadi

**Affiliations:** a Department of Neurosurgery, Nasser Institute for Research and Treatment, Cairo, Egypt; b Department of Neurology and Neurosurgery, Faculty of Medicine, Al-Azhar University, Cairo, Egypt; c Department of Health Professions, Faculty of Health Sciences, American University of Beirut, Beirut, Lebanon; d Department of Human Physiology and Basic Medical Sciences, Faculty of Medicine, Al-Azhar University, Cairo, Egypt; e Department of Neurosurgery, Misr University for Science and Technology, Giza, Egypt; f Department of Infectious Diseases and Tropical Medicine, Faculty of Medicine, Al-Azhar University, Cairo, Egypt

**Keywords:** Whipple's disease, CNS bacterial infection, Oculomasticatory myorhythmia, Case report

## Abstract

Whipple's disease, caused by the gram-positive actinomycete *Tropheryma whipplei*, is a rare chronic systemic illness with significant diagnostic and therapeutic challenges, particularly when the CNS is involved. This case report details a 46-year-old man presenting with a constellation of symptoms including fatigue, hypersomnia, weight loss, bifrontal headaches, abdominal pain, treatment-unresponsive diarrhea, and skin hyperpigmentation. Neurological examination revealed oculomasticatory myorhythmia, and imaging studies showed nodular enhancement of the hypothalamus and basal ganglia, along with retroperitoneal lymphadenopathy. Diagnosis was confirmed through duodenal biopsy with PAS staining and PCR analysis. The patient was successfully treated with a combination of intravenous ceftriaxone followed by oral doxycycline and hydroxychloroquine, resulting in significant symptom improvement and full recovery of neurological function. This case underscores the importance of early, aggressive treatment and provides a comprehensive review of the current literature on Whipple's disease with CNS involvement, including a comparison of treatment regimens and outcomes.

## Introduction

Whipple's disease is a rare chronic systemic illness caused by the gram-positive actinomycete *Tropheryma whipplei*. Although this pathogen is ubiquitous in the environment and can be asymptomatic in many carriers, Whipple's disease remains extremely uncommon, with an incidence of less than 1 per 1,000,000 people annually [1]. It predominantly affects males (8:1 ratio) and is typically diagnosed in the fifth to sixth decade of life, though it can occur at any age [2,3]. The pathogenesis of Whipple's disease involves host immune deficiencies, particularly in T cell responses. Studies have shown that Whipple's disease patients exhibit reduced or absent *T. whipplei*-specific Th1 responses in both peripheral and mucosal lymphocytes, while maintaining reactivity to other common antigens [4]. This impaired Th1 response is characterized by decreased production of IL-12 and IFN-gamma, accompanied by increased IL-4 secretion [5]. Additionally, regulatory T cells are elevated in the duodenal mucosa and peripheral blood of patients, further suppressing T cell activity [6]. These immune defects may enable *T. whipplei* to survive and replicate, leading to chronic infection in susceptible individuals.

The disease often begins in the small intestine and mesenteric lymph nodes, leading to symptoms such as malabsorption, diarrhea, weight loss, arthralgia, fever, and generalized lymphadenopathy. Due to its nonspecific initial presentation, Whipple's disease is frequently misdiagnosed as other rheumatic or gastrointestinal disorders [7]. CNS involvement in Whipple's disease can manifest in several ways: isolated CNS infection (fewer than 5%), classic Whipple's disease with CNS involvement (up to 50%), or CNS relapse after initial treatment [8,9]. The pathogenesis involves the presence of *T. whipplei* in the brain, forming nodules containing Sieracki cells and bacteria in various stages of degeneration [10]. CNS lesions commonly affect regions such as the periaqueductal gray matter, thalamus, hypothalamus, hippocampus, cingulate gyrus, basal ganglia, and cerebellum. Neurological symptoms can include ataxia, clonus, dementia, seizures, and frontal release signs. The hallmark findings in the CNS include complete vertical ophthalmoplegia, approximately 1 Hz convergent-divergent eye oscillations, and contractions of the masticatory muscles [11,12]. Diagnosis is typically confirmed by identifying PAS-positive foamy macrophages in a small bowel biopsy or by PCR detection of the bacterium [13,14].

## Case presentation

**Fig. 1:**
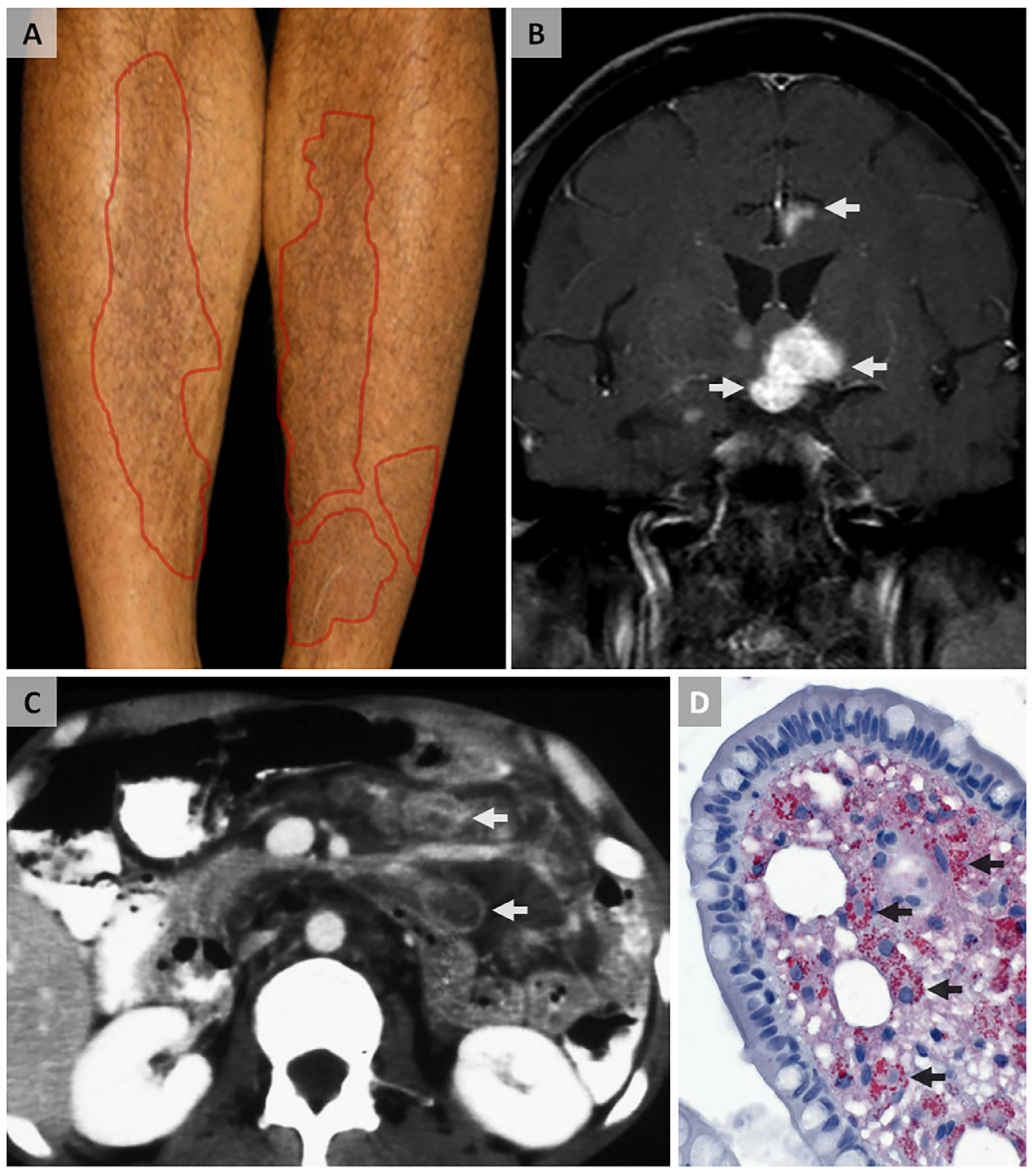
**Clinical and radiological findings in Whipple's disease with neurological involvement.** (**A**) A picture of the patient's exposed legs shows scattered hyperpigmented papules mainly below the knee (*markings*). (**B**) Coronal postcontrast brain T1WI sequence through the hypothalamus exhibits left-predominant nodular enhancement of the hypothalamus, inferior basal ganglia, and cingulum (*arrows*). (**C**) Axial postcontrast abdominal CT shows nonenhancing, hypoattenuated lymph nodes within the small-bowel mesentery (*arrows*). (**D**) Duodenal biopsy specimen reveals an infiltration of the lamina propria with macrophages containing PAS-positive inclusions (*arrows*).

**Fig. 2:**
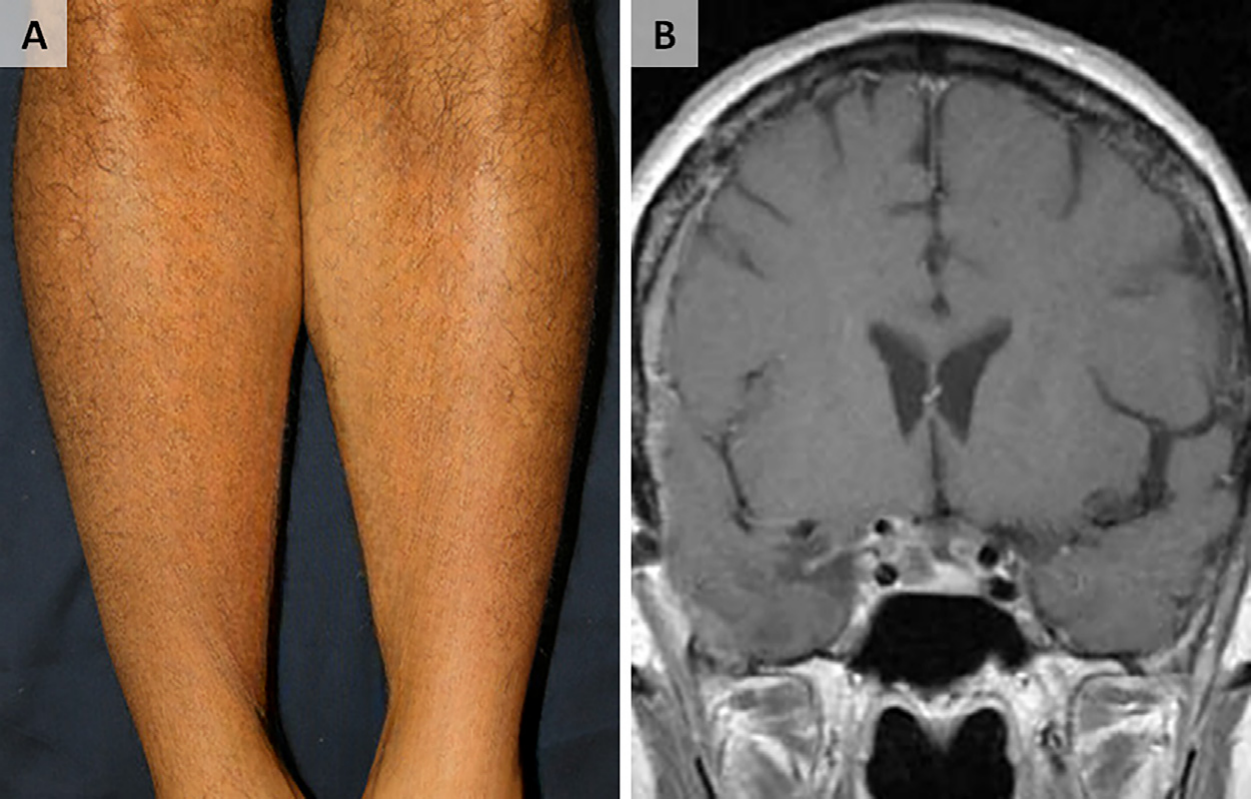
**Complete resolution of dermatological and CNS lesions following antimicrobial therapy.** (**A**) Clinical image of the patient's legs, taken 11 months after initiating therapy, showing complete resolution of the previously described papules. (**B**) Coronal postcontrast T1-weighted MRI sequence, demonstrating complete resolution of the previously noted brain lesions 11 months after starting antimicrobial therapy.

A 46-year-old man presented to the neurology clinic in August 2021 with a 6-month history of fatigue, hypersomnia, weight loss, and bifrontal headaches. He also reported episodes of abdominal pain and treatment-unresponsive diarrhea since January 2021, along with recurrent intermittent fleeting-type pain in multiple large joints and skin hyperpigmentation in the lower extremities (Fig. 1A). He denied any history of known cancers or genetic disorders. Clinical examination revealed smooth, continuous, slow (1-3 Hz), pendular, convergent-divergent nystagmus, concurrent contractions of the masticatory muscles, supranuclear vertical gaze palsy, and occasionally rhythmic movements of the limbs, consistent with oculomasticatory myorhythmia. Mild epigastric tenderness was noted on abdominal examination, and routine hematological and CSF studies demonstrated mild lymphocytic pleocytosis. Brain MR imaging showed left-predominant nodular enhancement of the hypothalamus, inferior basal ganglia, and the cingulum (Fig. 1B). Abdominal CT revealed retroperitoneal lymphadenopathy (Fig. 1C), and upper endoscopy with duodenal biopsy showed infiltration of the lamina propria with macrophages containing PAS-positive inclusions (Fig. 1D). CT scans of his chest and pelvis were unremarkable, and transesophageal echocardiography showed normal findings. Based on these findings, the diagnosis of Whipple's disease with CNS involvement was made. PCR-based analysis of the patient's saliva, stool, and CSF samples confirmed the diagnosis, and the baseline (pretreatment) titer of *T. whipplei* was determined. The patient was started on IV ceftriaxone (2g q12h) for 1 month, followed by oral doxycycline (100mg q12h) plus hydroxychloroquine (200mg q8h) for 1 year. Symptoms began to improve within 2 weeks of initiating treatment, and full recovery of his neurological and dermatological complications was achieved after 11 months (Fig. 2).

## Discussion

This case exemplifies the classical manifestations of Whipple's disease with CNS involvement whereby the patient exhibited a constellation of symptoms including hypersomnia, weight loss, bifrontal headaches, and abdominal issues, alongside distinctive neurological signs such as oculomasticatory myorhythmia, nystagmus, and supranuclear gaze palsy. These symptoms align with previously documented cases where neurological manifestations are a critical aspect of the disease presentation [[8], [9], [10], [11], [12], [13], [14]]. The patient's skin hyperpigmentation, joint pain, and gastrointestinal symptoms are consistent with the systemic nature of Whipple's disease [7].

Oculomasticatory myorhythmia is a rare but classic pathognomonic manifestation of CNS involvement in Whipple's disease. It is characterized by rhythmic contractions of the masticatory muscles and eye movements, a hallmark feature that can assist in differentiating Whipple's disease from other neurological disorders [11,12,15]. While Louis *et al.* [16] reported this symptom in ∼20% of CNS cases, it typically appears in later stages of the disease. Interestingly, our patient exhibited this sign relatively early, which may have contributed to the timely diagnosis. Additionally, the brain MRI in our case revealed nodular enhancement in the hypothalamus, basal ganglia, and cingulum—findings that, while partially consistent with Black *et al.* [17], included the involvement of the cingulum, an unusual site noted in only a few cases.

When considering differential diagnoses, paraneoplastic limbic encephalitis, particularly associated with anti-Ma2 antibodies, can display similar imaging features; however, the clinical presentation is distinct. Additionally, this patient had no history of cancer, which excludes metastasis for the same reasons. Pilomyxoid astrocytoma can also have comparable imaging findings but usually presents earlier in life and lacks systemic manifestations.

A review of the literature and our institute's clinical database reveals a variety of CNS manifestations in Whipple's disease, including ataxia, seizures, dementia, and other focal neurological deficits [[8], [9], [10], [11], [12], [13], [14], [15], [16]]. These manifestations are typically associated with specific MRI findings, such as enhancement or lesions in the midbrain, hypothalamus, mesial temporal lobes, and corticospinal tracts, though it can also be normal [17]. Table 1 summarizes cases from our institute's database, providing a comprehensive view of the diversity in clinical presentations and imaging findings associated with Whipple's disease with CNS involvement.

**Table 1:** Overview of Whipple's disease cases with central nervous system (CNS) involvement from our clinical database.

Age	Sex	Initial symptoms	Neurological symptoms	CNS imaging findings	Treatment and outcome
52	M	Diarrhea, weight loss, abdominal pain	Ataxia, dementia	Hypothalamic lesions	Ceftriaxone followed by doxycycline; significant improvement
45	F	Joint pain, fever, weight loss	Oculomasticatory myorhythmia	Basal ganglia enhancement	Penicillin followed by doxycycline; full recovery
60	M	Diarrhea, arthralgia	Seizures, ataxia	Thalamic lesions	Ceftriaxone followed by doxycycline; partial improvement
40	M	Abdominal pain, skin pigmentation	Frontal lobe symptoms	Cingulate gyrus involvement	Ceftriaxone followed by doxycycline and hydroxychloroquine; full recovery
49	M	Weight loss, diarrhea	Dementia, clonus	Hippocampal lesions	Ceftriaxone followed by doxycycline; significant improvement
53	F	Diarrhea, joint pain	Oculomasticatory myorhythmia	Cerebellar involvement	Penicillin followed by doxycycline; full recovery
58	M	Abdominal pain, fever	Gait ataxia, nystagmus	Hypothalamic and basal ganglia lesions	Ceftriaxone followed by doxycycline; significant improvement
62	M	Weight loss, abdominal pain	Vertical gaze palsy, dementia	Periaqueductal gray matter lesions	Ceftriaxone followed by doxycycline; partial improvement
54	F	Skin pigmentation, diarrhea	Myoclonus, tremors	Thalamic and basal ganglia lesions	Ceftriaxone followed by doxycycline and hydroxychloroquine; full recovery
51	M	Joint pain, abdominal pain	Seizures, ataxia	Lesions in thalamus and basal ganglia	Ceftriaxone followed by doxycycline; significant improvement
47	F	Weight loss, fever	Oculomasticatory myorhythmia	Hippocampal lesions	Ceftriaxone followed by doxycycline; full recovery
60	M	Diarrhea, abdominal pain	Ataxia, cognitive decline	Cingulate gyrus involvement	Ceftriaxone followed by doxycycline; significant improvement
55	M	Joint pain, weight loss	Dementia, tremors	Basal ganglia lesions	Ceftriaxone followed by doxycycline and hydroxychloroquine; full recovery
49	F	Abdominal pain, fever	Ataxia, myoclonus	Hippocampal and cerebellar lesions	Ceftriaxone followed by doxycycline; partial improvement
63	M	Weight loss, skin pigmentation	Seizures, clonus	Periaqueductal gray matter lesions	Ceftriaxone followed by doxycycline and hydroxychloroquine; full recovery

The table presents a summary of key cases of Whipple's disease with CNS involvement from our institute's clinical records. For each case, details are provided regarding the patient's age, sex, initial symptoms, neurological presentation, CNS imaging findings, diagnostic techniques, treatment regimens, and clinical outcomes. This compilation emphasizes the heterogeneity in clinical features, the diverse diagnostic methods utilized, and the varying responses to treatment among patients with CNS involvement in Whipple's disease.

The management of Whipple's disease with CNS involvement typically involves a 2-phase approach. Initial treatment usually consists of intravenous antibiotics, such as ceftriaxone or penicillin. This intensive regimen is aimed at rapidly achieving high drug concentrations to eradicate *T. whipplei.* Studies have demonstrated that IV ceftriaxone is particularly effective due to its good CNS penetration and broad-spectrum activity [18,19]. The duration of IV therapy usually ranges from 2 to 4 weeks, depending on the severity of the disease and the clinical response. This is followed by oral trimethoprim-sulfamethoxazole (TMP-SMX), or doxycycline combined with hydroxychloroquine for maintenance therapy. While earlier studies suggested a 12-month course of TMP-SMX [18], more recent evidence indicates that a 3-month regimen may be equally effective [20]. However, CNS involvement can occur during or after TMP-SMX treatment, necessitating close monitoring [21]. Doxycycline is preferred for its excellent oral bioavailability and prolonged tissue penetration, which helps in eliminating any residual bacterial load and preventing relapse [22]. Hydroxychloroquine, an antimalarial with anti-inflammatory properties, is sometimes added to enhance the effectiveness of the treatment, particularly in cases with persistent or severe symptoms [22].

Recent studies on Whipple's disease treatment, particularly for cases with central nervous system involvement, suggest that combination therapy is more effective than monotherapy. Lagier *et al.* [22] found that doxycycline and hydroxychloroquine led to better outcomes compared to trimethoprim/sulfamethoxazole. Compain *et al.* [21] reported favorable outcomes in 78% of patients using various antibiotic combinations, including hydroxychloroquine. However, Schnider *et al.* [19] observed that 40% of patients treated with trimethoprim/sulfamethoxazole did not respond, recommending ceftriaxone and streptomycin as initial treatment. Hyche *et al.* [23] described a case where ceftriaxone followed by doxycycline and hydroxychloroquine initially improved symptoms, but progress later slowed. These findings highlight the complexity of treating Whipple's disease with CNS involvement and suggest that early initiation of combination therapy, including intravenous antibiotics followed by oral doxycycline and hydroxychloroquine, may lead to improved outcomes, a hypothesis supported by our case and several studies have documented the success of this approach as well as regard to the following clinical aspects [[18], [19], [20], [21], [22], [23]]:

• Rapid Clinical Improvement: Patients typically show substantial clinical improvement within weeks of starting combination therapy. Early initiation of this comprehensive treatment regimen correlates with faster resolution of both systemic and neurological symptoms, compared to treatment regimens that delay or omit the use of IV antibiotics.
• Reduced Risk of Relapse: Early and aggressive treatment helps in reducing the risk of disease relapse, a common issue in Whipple's disease. Prolonged oral therapy ensures that any residual bacteria are effectively eradicated, thereby lowering the chances of recurrence.
• Long-term Outcomes: Long-term follow-up studies have shown that patients who receive prompt and appropriate combination therapy often achieve full recovery of neurological function and maintain improved quality of life. Delayed treatment or incomplete courses of therapy can result in suboptimal outcomes and persistent symptoms.
• Comparison with Historical Treatments: Historical data comparing earlier treatment approaches with more modern combination therapies highlight the advantages of the current regimen. For example, prior regimens that relied solely on IV antibiotics or shorter courses of oral antibiotics often resulted in higher relapse rates and less favorable outcomes.

To sum up, this case report and review emphasize the critical role of early and aggressive treatment in managing Whipple's disease with CNS involvement. Combination therapy starting with IV antibiotics followed by oral doxycycline, and in some cases, hydroxychloroquine, leads to significantly better outcomes. The included case series highlights the variability in treatment regimens and outcomes, underscoring the need for tailored approaches to optimize patient care. Future research should focus on refining treatment protocols and improving diagnostic methods to enhance early detection and management of this challenging disease.

## Methods

The work presented in this manuscript has been reported in line with the SCARE criteria [24].

## Patient consent

The authors declare that they have obtained consent from the patient.

## Ethical approval

The ethics committee at Al-Azhar University hospitals officially approved all the performed procedures and the followed guidelines under the reference number of HSZ-21-000014576.
